# Greater muscle mass does not affect peripheral hypercapnic chemosensitivity during exercise in healthy adults

**DOI:** 10.14814/phy2.71102

**Published:** 2026-09-09

**Authors:** Armando J. Aguero, Connor J. Doherty, Paolo B. Dominelli

**Affiliations:** ^1^ Department of Kinesiology University of Waterloo Waterloo Ontario Canada; ^2^ Faculty of Kinesiology University of Calgary Calgary Alberta Canada

**Keywords:** hypercapnia, peripheral chemoreceptors, ventilation

## Abstract

Peripheral hypercapnic chemosensitivity (PHC) is the ventilatory response to a brief rise in arterial carbon dioxide tension. PHC is known to increase at the onset of exercise, with no further increases with progressive exercise intensity. The mechanisms for this augmentation remain unclear but may involve central command. We examined whether PHC scales with active muscle mass by comparing small‐muscle (rhythmic handgrip, RHG), intermediate‐muscle (single‐leg extension, SLE), and large/whole‐body (cycling, BIKE) exercise. Twenty‐two healthy adults (*n* = 11 females) performed RHG, SLE, and BIKE exercise. PHC was measured before and during exercise using four trials, each consisting of two inhalations of 10% carbon dioxide. All exercise modalities produced a significant increase in PHC from rest. During exercise, SLE and BIKE (0.895 ± 0.388 and 1.028 ± 0.460 L·min^−1^·mmHg^−1^, respectively) elicited significantly greater PHC than RHG (0.683 ± 0.247 L·min^−1^·mmHg^−1^) (*p* = 0.037 and 0.015, respectively), while SLE and BIKE were not different (*p* = 0.263). Exercise sensitizes PHC regardless of recruited muscle mass, but augmentation does not scale linearly with muscle mass. As PHC increased with recruitment up to SLE, with no further increase during BIKE. These findings suggest that the augmentation of PHC is more closely associated with the transition to exercise, although the present exploratory study cannot determine the mechanism(s) responsible.

## 1 INTRODUCTION

Peripheral hypercapnic chemosensitivity (PHC) is the rapid ventilatory response elicited by a transient rise in partial pressure of arterial carbon dioxide (PaCO_2_) (Forster et al., 2012; O'Regan & Majcherczyk, 1982). This response is peripherally mediated, as transient PaCO_2_ changes are too brief to stimulate central chemoreceptors (McClean et al., 1988). Peripheral hypercapnic chemosensitivity is often quantified using a transient hypercapnic trial involving one to three breaths of CO_2_‐enriched gas (10%–13%), with the change in inspired ventilation (ΔV̇_I_) relative to the change in end‐tidal CO_2_ (ΔP_ET_CO_2_) yielding PHC in L·min^−1^·mmHg^−1^ (McClean et al., 1988). PHC rises at the onset of low‐intensity whole‐body exercise (Chang et al., 2025; Forster et al., 2012; Granger et al., 2020; Levitzky, 1986; Mann et al., 2022; Pianosi & Khoo, 1995; Thompson et al., 2024; Wright et al., 2024); however, plateaus with further workload increases (Mann et al., 2024; Thompson et al., 2024; Thompson et al., 2025; Wright et al., 2024). Central command is the feedforward mechanism that contributes to cardiovascular and ventilatory control (Fadel & Fadel, 2015; Williamson, 2010; Williamson et al., 2006; Wright et al., 2024). For example, it drives anticipatory increases in heart rate and ventilation before exercise (Miyamoto et al., 2022; Williamson, 2010). Central command has been shown to modulate the arterial baroreflex during exercise, as both cardiac and sympathetic baroreflex sensitivities are reduced during voluntary movement (Doherty et al., 2018; Fadel & Raven, 2012). Lastly, central command shows a dose‐dependent increases in heart rate and mean arterial pressure through greater exercise intensity and muscle recruitment, progressing from isolated small muscle groups to combined limb exercise (Mitchell et al., 1980; Williamson et al., 2006). In addition, afferent mechanoreceptor feedback from contracting skeletal muscle contributes to the increase in heart rate during passive, non‐volitional dorsiflexion (Gladwell & Coote, 2002), and increases in minute ventilation at the onset of rhythmic passive leg extension (Morikawa et al., 1989). For the present exploratory study, a low‐intensity exercise was selected to ensure PHC sensitization while minimizing activation of the muscle metaboreflex. Although it is not possible to fully differentiate between central command and mechanoreceptor feedback during voluntary exercise, this approach allows for a comparison of differences in active muscle mass (Nóbrega et al., 1994). While whole‐body exercise such as cycling (BIKE) is known to increase PHC, it remains unknown whether smaller muscle mass exercises such as rhythmic handgrip (RHG) or single‐leg extension (SLE) elicit comparable PHC responses. Moreover, it is unclear whether different levels of activation of central command itself proportionally enhance PHC sensitivity.

The purpose of the present exploratory study was to examine the change in PHC with different levels of central command and muscle afferent feedback achieved through varying amounts of active muscle mass. We hypothesized that increases in PHC would scale with the volume of active muscle mass, such that RHG would produce the smallest measurable augmentation, whereas SLE and BIKE would evoke progressively greater increases in PHC.

## 2 METHODS

### 2.1 Ethical approval

The experimental procedures were approved by the University of Waterloo Clinical Research Ethics Board (#46216). Protocols adhered to the *Declaration of Helsinki* (except database registration), and participants provided written informed consent.

### 2.2 Participants

Twenty‐two (*n* = 11 females) individuals completed a single day of testing. All participants were healthy young adults who exercised >3 times per week for a total of >150 min. Participants were free from metabolic, respiratory or cardiovascular conditions, were non‐smokers and non‐obese (BMI <30 kg/m^2^). Females using progesterone‐only birth control were excluded due to its hypercapnic effects (Smith & Mines, 1982). Menstrual cycle phase was not tracked or controlled for; as menstrual phase does not impact the ventilatory response to exercise (MacNutt et al., 2012; Mann et al., 2024). Participants were recruited through convenience sampling at the University of Waterloo. Participants abstained from exhaustive exercise for 24 h, and alcohol and caffeine for 12 h pre‐testing.

### 2.3 Experimental overview

**FIGURE 1:**
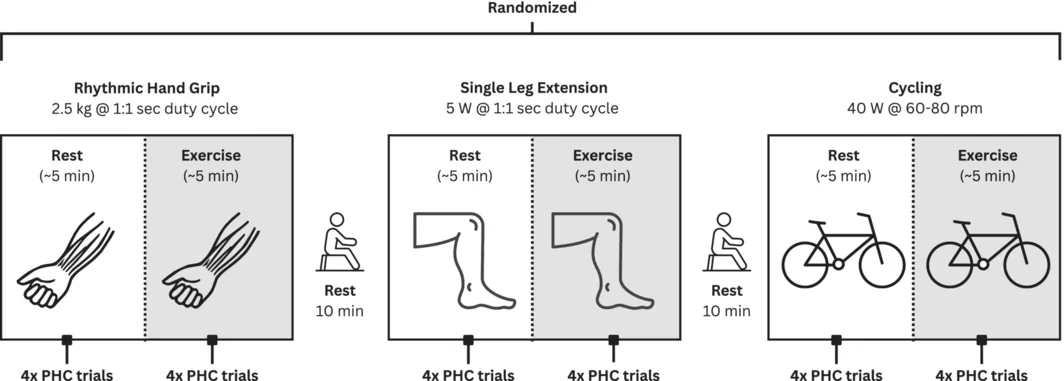
Experimental overview: single testing session. Three exercises are randomized a PHC trial: Peripheral Hypercapnic Chemosensitivity (PHC), Revolutions per minute (rpm), Watts (W), *N* = 22.

A detailed schematic of the study protocol can be found in Figure 1. Participants completed a single testing session to evaluate PHC during three exercise modalities (RHG, SLE, and BIKE), spanning a range of muscle mass. Modalities were randomized to minimize carryover effects, >10 min of rest between each modality for standardization and re‐instrumentation. Each modality consisted of a resting and an exercise condition, each lasting approximately 7 min (14 min total per modality). Four PHC trials were completed during each condition.

### 2.4 Peripheral hypercapnic chemosensitivity testing

Each peripheral hypercapnic chemosensitivity trial consisted of two inhalations of a hypercapnic gas mixture (10% CO_2_, 21% O_2_, balance N_2_) from a pre‐filled Douglas bag (Mann et al., 2022; Thompson et al., 2024; Wright et al., 2024). The stimulus is delivered through a silent manual three‐way valve that was open to room air and momentarily switched to the hypercapnic mixture for two inhalations. Within each condition, four PHC trials were completed, yielding a total of eight trials per modality. Prior to the first stimulus in every condition, ~120 s of data were collected to determine pre‐stimulus values for V̇_I_, P_ET_CO_2_, end‐tidal O_2_ (P_ET_O_2_), and breathing frequency (F_b_). Each subsequent trial was performed after inspired ventilation (V̇_I_) and end‐tidal CO_2_ (P_ET_CO_2_) returned to the pre‐stimulus baseline, taking approximately 45–60 s. No visual, verbal, or audible cues signaled stimulus onset or offset; investigators used real‐time continuous measurements of ventilatory flow to switch the valve.

### 2.5 Exercise testing

Three exercise modalities were used to engage varying amounts of muscle mass. Each exercise modality lasted ~14 min including the resting baseline and active PHC trials. The RHG was conducted on a custom handgrip device that was adjusted to be level with the participant's elbow while sitting upright. The device is comprised of two parallel grasp bars mounted on an adjustable tripod: (one stationary, one moving 4–8 cm). A 2.5 kg mass was suspended from the mobile bar to a pulley. Exercise was performed with a 1:1 s duty cycle (1 s contraction, 1 s relaxation), at a cadence of 30 contractions per minute.

The SLE was performed in an upright‐seated position with the right leg as the active limb. A custom ergometer consisting of a cycle ergometer frame (Model 868; Monark, Varberg, Sweden) that provided a constant 5 W of resistance (Doherty et al., 2025). A rod mounted to the pedal held a kicking pad, which was positioned below the mid‐shin. Contractions were then executed at a 1:1 duty cycle for 30 contractions per minute.

Participants completed the BIKE condition on a cycle ergometer (Monark LC7TT, Vansbro, Sweden). Participants completed the exercise at a resistance of 40 W and at a self‐selected cadence between ~60–80 revolutions per minute.

### 2.6 Data collection

Raw data was collected and stored at 200 Hz using a 16‐channel analog‐to‐digital acquisition system (PowerLab/16SP model ML 795; AD Instruments, Colorado Springs, CO). Inspired flow was measured using an unheated pneumotachometer (model 3813; Hans Rudolph, Kansas City, MO), calibrated using a 3 L syringe. An oro‐nasal mask was used with a two‐way non‐rebreathing valve (Model 2700, Hans Rudolph, Kansas City, MO). The valve was connected with large‐bore tubing to the gas reservoir. A sample line from the mask was connected to calibrated O_2_ and CO_2_ analyzers (AEI Technologies S‐3‐A/I and CD‐3Am, respectively; Applied Electrochemistry, Bastrop, TX) to collect P_ET_CO_2_ and P_ET_O_2_. For the final minute of each condition, the sample line was switched to a 5 L mixing chamber to allow for the measurement of mixed expired gases for the calculation of V̇O_2_ and V̇CO_2_. Heart rate was measured using a Polar heart rate monitor (Model T34; Polar Electro Oy, Kempele, Finland).

### 2.7 Data analysis

Peripheral hypercapnic chemosensitivity for each two‐breath CO_2_ trial was quantified as the ratio of the change in V̇_I_ to the related change in P_ET_CO_2_: PHC (L·min^−1^·mmHg^−1^) = (V̇_I peak_ − V̇_I baseline_)/(P_ET_CO_2,peak_ − P_ET_CO_2,baseline_) (Mann et al., 2022; Thompson et al., 2024; Wright et al., 2024). Prior to every trial a pre‐stimulus baseline of V̇_I_ and P_ET_CO_2_ was determined by using the 20 s immediately prior to the stimulus. The peak responses for V̇_I_ were identified as the single highest tidal value recorded within 30 s after the last inhalation of the hypercapnic stimulus. The peak P_ET_CO_2_ was identified as the highest end‐expiratory CO_2_ pressure reached within the same interval. Gas traces were temporally aligned with the flow signals to account for analyzer delay, ensuring that P_ET_CO_2_ peak corresponded to the end of expiration.

Four PHC trials were performed in each condition. Any trial that contained a breath that substantially deviated from normal tidal breathing (e.g., coughs, sneezes, yawns, sighs, or speech) were excluded from analysis to ensure representative baseline and peak ventilatory responses. All valid trials were averaged to yield a single PHC value for each condition (e.g., RHG‐rest, RHG‐active), when all four trials were acceptable, all four were averaged; otherwise, the remaining three valid trials were averaged. A minimum of three valid trials was required for analysis.

Raw flow signals were numerically integrated to yield volume, permitting the breath‐by‐breath calculation of tidal volume (V_t_) and F_b_. Using V_t_ and F_b_, V̇_I_ was calculated as their product and expressed as in body temperature pressure saturated.

### 2.8 Statistical analysis

All statistical analyses were performed using GraphPad Software (GraphPad Prism 10.4.1, La Jolla, CA). Sphericity was assessed using Mauchly's test, and Greenhouse–Geisser corrections were applied whenever the epsilon was greater than 0.75. Ventilatory and cardiovascular variables and their derived responses were examined with a two‐way repeated‐measures ANOVA (Modality: RHG, SLE, BIKE × Condition: Rest, Active). The absolute change and percent change in PHC from baseline were each assessed with a one‐way repeated‐measures ANOVA (factor: Modality). Tukey's post hoc comparisons were performed on significant interactions where appropriate. Age, height, mass, body mass index, and body surface area were compared between males and females using independent samples *t*‐tests. Statistical significance was set at *p* < 0.05 for all tests, and adjusted *p* values are reported where applicable. Data are presented as mean ± standard deviation.

## 3 RESULTS

### 3.1 Demographics

Participants were 24 ± 3 years of age, with a mean height of 172 ± 9 cm, body mass of 70 ± 13 kg, body mass index of 23.2 ± 3.2 kg/m^2^, and body surface area of 1.8 ± 0.2 m^2^. Males and females did not differ in age (23 ± 3 vs. 24 ± 3 years) or body mass index (23.5 ± 3.7 vs. 23.0 ± 2.6 kg/m^2^). Males were significantly taller than females (179 ± 5 vs. 165 ± 6 cm, *p* < 0.001) and had a greater body surface area (1.9 ± 0.2 vs. 1.7 ± 0.2 m^2^, *p* = 0.011). Body mass was 76 ± 13 kg in males and 63 ± 10 kg in females.

### 3.2 Ventilatory and heart rate response at rest and during exercise

**FIGURE 2:**
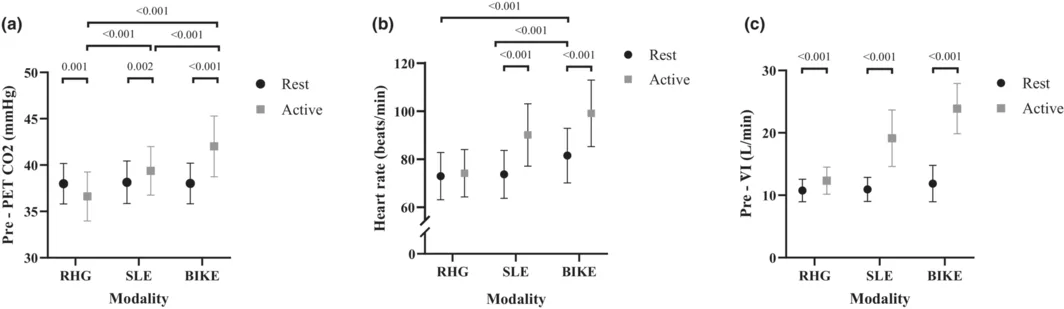
(a) Heart rate data from rest to exercise for rhythmic handgrip (RHG), single‐leg extension (SLE), and cycling (BIKE). (b) Pre‐stimulus end‐tidal CO_2_ (Pre‐P_ET_CO_2_) values recorded immediately before each transient CO_2_ stimulus. (c) Pre‐stimulus volume inspired (V̇_I_) for each individual participant (paired lines) at rest and during the active condition. Data are presented as mean ± SD (*N* = 22). Statistical comparisons were performed using a one‐way repeated‐measures ANOVA with Tukey's post hoc test where appropriate.

Ventilatory values for rest and exercise conditions are summarized in Table 1; cardiovascular and ventilatory data are shown in Figure 2. During exercise, SLE and BIKE elicited higher HR, pre‐V̇_I_, pre‐P_ET_CO_2_ (Figure 1a–c), Vt, as well as larger ΔV̇_I_ and ΔP_ET_CO_2_ than their respective resting conditions (all *p* < 0.001, except for pre‐PETCO_2_ during SLE, which increased from rest to active exercise at *p* = 0.002). During exercise, RHG increased pre‐V̇_I_ (*p* < 0.001) and ΔV̇I (*p* = 0.005), whereas pre‐P_ET_CO_2_ decreased (*p* = 0.001) compared to the resting condition. While, HR, V_t_, and ΔP_ET_CO_2_ were unchanged. At rest, HR was greater during BIKE than during both RHG and SLE (both *p* < 0.001), whereas RHG and SLE did not differ (*p* = 0.809). Heart rate, V_t_, pre‐V̇_I_, pre‐P_ET_CO_2_, ΔV̇_I_ and ΔP_ET_CO_2_ each displayed significant main effects of modality and condition and a significant modality × condition interaction (all *p* < 0.001). Breathing frequency rose in the active condition of each task compared to their respective resting condition (*p* < 0.05); the main effect of condition and the interaction were significant (*p* < 0.001) whereas the main effect of modality was not (*p* = 0.086).

**TABLE 1:** Cardiopulmonary responses to CO_2_ testing during different exercise modalities.

	Rhythmic hand grip		Single leg extension		Bike		Modality	Condition	Interaction
	Rest	Active	Rest	Active	Rest	Active			
HR, beats/min	73 ± 10	74 ± 10	74 ± 10.0	90 ± 13†	82 ± 12	99 ± 14†	<0.001	<0.001	<0.001
V̇CO_2_, L/min	0.3 ± 0.1	0.3 ± 0.1	0.3 ± 0.1	0.5 ± 0.1†	0.3 ± 0.1	0.8 ± 0.2†	<0.001	<0.001	<0.001
V̇O_2_, L/min	0.3 ± 0.1	0.3 ± 0.1	0.3 ± 0.4	0.7 ± 0.8†	0.3 ± 0.1	1.1 ± 0.9†	<0.001	<0.001	<0.001
F_b_, breaths/min	13.7 ± 4.7	15.3 ± 3.7*	12.8 ± 4.4	18.1 ± 5.2†	13.2 ± 4.4	16.3 ± 5.0*	0.086	<0.001	<0.001
V_t_, L	0.8 ± 0.3	0.8 ± 0.4	0.8 ± 0.3	1.1 ± 0.4†	1.0 ± 0.4	1.6 ± 0.5†	<0.001	<0.001	<0.001
V̇_I_, L/min	10.9 ± 1.7	12.5 ± 2.0†	10.8 ± 2.2	20.2 ± 4.7†	12.8 ± 3.0	26.3 ± 4.5†	<0.001	<0.001	<0.001
ΔV̇_I_, L/min	4.7 ± 2.0	5.8 ± 3.9*	5.1 ± 2.8	9.5 ± 4.3†	6.2 ± 3.4	13.4 ± 5.2†	<0.001	<0.001	<0.001
Δ P_ET_CO_2_, mmHg	8.6 ± 2.0	8.9 ± 2.2	8.7 ± 2.5	11.2 ± 3.3†	8.6 ± 2.6	13.4 ± 2.3†	<0.001	<0.001	<0.001
PHC CV, %	32.4 ± 15.5	31.0 ± 18.4	32.2 ± 14.5	22.0 ± 12.4	32.7 ± 27.1	19.8 ± 8.4	0.343	0.016	0.137

*Note*: *p < 0.05 and †*p* < 0.001 rest vs. active within modality.

Abbreviations: CO_2_, carbon dioxide; CV, coefficient of variationFb, breathing frequency; HR, heart rate; V̇CO_2_, carbon dioxide production; V̇_I_, inspired ventilation; V̇O_2_, oxygen uptake; Vt, tidal volume; ΔP_ET_CO_2_, change in end‐tidal carbon dioxide; ΔV̇_I_, change in inspired ventilation.

### 3.3 Effect of exercise modality on the hypercapnic ventilatory response

**FIGURE 3:**
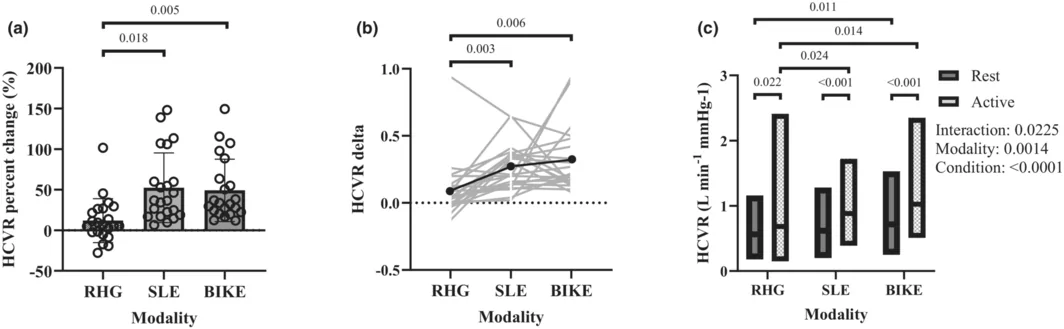
(a) The percent change in peripheral hypercapnic chemosensitivity (PHC) during the active condition for (RHG), single‐leg extension (SLE), and cycling (BIKE). (b) The absolute change in PHC (active minus rest) across modalities. (c) The rest and exercise PHC for each modality. Data in Panel c is represented box plot showing the median (horizontal line) and minimum and maximums. All figures represent an *N* = 22. Statistical comparisons were performed using a one‐way repeated‐measures ANOVA with Tukey's post hoc test where appropriate.

Figure 3 presents PHC values, and absolute and relative change in PHC from rest to exercise in each modality. Absolute PHC showed a main effect of modality (*p* = 0.001), condition (*p* < 0.001), and a modality × condition interaction (*p* = 0.023). Each modality exhibited a significant increase in PHC from rest to active (BIKE and SLE, *p* < 0.001; RHG *p* = 0.022). At rest, BIKE elicited a greater PHC than RHG (*p* = 0.011), while RHG and SLE did not differ (*p* = 0.526), nor did SLE and BIKE (*p* = 0.135). During exercise, both BIKE and SLE were greater than RHG (*p* = 0.014 and *p* = 0.024, respectively). The significant modality × condition was driven by a large rise in PHC in SLE and BIKE compared to the small rise in RHG when going from rest to active. The percent change in PHC from rest to active is shown in Figure 3a, where both BIKE and SLE had a greater increase over RHG (*p* = 0.005 and *p* = 0.018, respectively), with SLE and BIKE not being different (*p* = 0.813). The same pattern emerged for the delta PHC (Figure 3b) with BIKE and SLE both exceeding RHG (*p* = 0.006 and *p* = 0.003, respectively), with no difference between SLE and BIKE (*p* = 0.883). In addition, both percent change and PHC delta showed a main effect of modality (*p* = 0.010 and *p* = 0.003, respectively), driven by RHG showing a smaller increase than SLE and BIKE, while SLE and BIKE did not differ. The trial‐to‐trial coefficient of variation for PHC is shown in Table 1. There was a significant main effect of condition (*p* = 0.016) with exercise being lower than rest but no main effect of modality (*p* = 0.354), nor was there an interaction (*p* = 0.127).

## 4 DISCUSSION

### 4.1 Major findings

The major finding of this exploratory study was an increase in PHC across all three exercise modalities from rest to active, supporting our hypothesis that exercise augments PHC even with small muscle mass. However, our hypothesis that PHC would scale progressively with active muscle mass was not supported. While PHC in the active condition during SLE and BIKE exceeded RHG, SLE and BIKE did not differ from each other despite BIKE recruiting substantially more muscle mass. The increase in PHC during whole‐body exercise has been consistently observed, but the change in PHC during smaller‐muscle mass exercise (RHG and SLE) is novel. Together, these findings indicate that PHC augmentation does not scale progressively with active muscle mass. Therefore, factors responsible for augmenting PHC are most strongly associated with the transition from rest to exercise rather than the amount of muscle mass recruited during exercise.

### 4.2 Peripheral hypercapnic chemosensitivity during exercise

We have recently been seeking to identify the mechanisms behind PHC sensitizations during exercise. Previously, we found that acute whole‐body exercise results in a 23%–102% increase in PHC compared to rest (Chang et al., 2025; Mann et al., 2022; Thompson et al., 2024; Wright et al., 2024). In the current study, our magnitude of increase was similar (Figure 3c) with an increase of 11.9% ± 27.0%, 52.4% ± 42.8%, and 49.2% ± 38.4% for RHG, SLE, and BIKE, respectively. We also demonstrated that the increase in PHC from rest to exercise is reproducible across multiple days and is unlikely to reflect testing variability (Thompson et al., 2025). We have also found that adding another peripheral ventilatory stimulant (hypoxia) further augmented the PHC during exercise. Although chronic hypoxia elevated resting and exercise PHC, exercise‐related sensitization was not different (Chang et al., 2025). As such, we concluded that the initiation of exercise increases PHC independently of the sensitization from chronic hypoxia. That chronic hypoxia and exercise independently influenced the PHC suggests different mechanisms play a role in the sensitization seen during exercise.

However, not all previous studies have reported a significant increase in chemoreceptor sensitivity at the onset of exercise. Using modified rebreathing techniques, Casey et al. (1987) assessed the central chemoreceptor response, and Duffin and McAvoy (1988) assessed the peripheral chemoreceptor response during the transition from rest to exercise. Neither study found a significant change in chemoreceptor threshold at the onset of exercise. The studies showed an ~8% increase in central chemoreceptor sensitivity and an ~8.5% increase in peripheral chemoreceptor sensitivity; however, these increases in chemosensitivity were not interpreted to be physiologically significant (Casey et al., 1987; Duffin & McAvoy, 1988). Differences in methodologies likely explain the discrepancies in studies. Previously, the initial transition from rest to exercise was measured using the third breath after the onset of exercise or using the early exercise response (Casey et al., 1987; Duffin & McAvoy, 1988) which captured different components of the exercise ventilatory response compared to our steady state testing. The neural inputs that are associated with exercise onset will strongly influence the ventilatory response, whereas the steady state response reflects the integrated effects of the exercise. Moreover, the rebreathing techniques used in these studies also differ substantially from the transient hypercapnic stimulus used in this study. A hyperoxic rebreathing technique was used to assess peripheral chemoreceptor activity while breathing 7% CO_2_; this method cannot exclude the ongoing activation of central chemoreceptors in mediating the ventilatory response, further confounding the interpretation of the role of peripheral chemoreceptors (Casey et al., 1987; Duffin & McAvoy, 1988). Therefore, the differences in chemoreceptor sensitivity due to the onset of exercise do not necessarily contradict the findings in the present exploratory study.

### 4.3 Impact of muscle mass on peripheral hypercapnic chemosensitivity

Mechanistically, we expected a larger active muscle mass to increase central command activation, thereby elevating ventilatory drive and cardiovascular control (Williamson et al., 2006). A small amount of muscle mass is recruited during RHG and evokes lower central motor outflow compared to SLE or BIKE, yielding a smaller rise in PHC from the respective rest period. Whereas SLE and BIKE recruit substantially more muscle and generate larger feedforward/feedback signals, thus the PHC was significantly greater (Figure 3). Together with our previous finding that PHC increases with the initiation of cycling but does not further increase with progressive exercise intensity, these results suggest that the factors responsible for augmenting PHC are most strongly associated with the transition from rest to exercise and do not continue to increase proportionally with either active muscle mass or exercise intensity (Wright et al., 2024). While the present study cannot identify the specific mechanism responsible for this response, the similar PHC observed during SLE and BIKE suggests that recruiting additional muscle mass beyond that required during SLE does not further augment PHC.

### 4.4 Possible reflex mechanisms: Central command

We hypothesized that central command may play a role in PHC sensitization. At rest, PHC did not differ between SLE and RHG when participants were seated in the same position. However, resting PHC during BIKE was greater than during RHG despite no exercise being performed (Figure 3c). The rise in resting PHC in anticipation of exercise was also observed previously; participants had a significantly higher resting PHC seated on a cycle ergometer than seated on a chair (Wright et al., 2024). We attributed this initial increase to an anticipatory response secondary to central command activation prior to exercise. With the increasing muscle mass during the exercising portion of the study, it is assumed that there is likewise an incremental increase in central command signaling. Although PHC increased from rest to exercise in all modalities, the increase was greater during SLE and BIKE than during RHG.

### 4.5 Afferent feedback

It is unlikely that Type IV afferents (chemoreceptors) can be attributed to the increase seen in PHC during the exercise. By design, all exercises were performed at a low intensity, so the metabolic stress accumulated was minimal. Prior studies have also shown that increases in metaboreceptor feedback do not increase PHC past the commencement of exercise up to and beyond the respiratory compensation point (Thompson et al., 2024; Thompson et al., 2025; Wright et al., 2024). Due to the prior studies and the low intensity, the increase from RHG to SLE and BIKE cannot reasonably be attributed to greater metaboreceptor afferent feedback from the exercising muscle. However, with the observed increase in PHC across a range of small to large muscle mass, the influence of type III muscle mechanoreceptor feedback cannot be overlooked. Mechanoreceptors respond to muscle stretch and contraction, augmenting sympathetic outflow and ventilatory drive early in exercise (Morikawa et al., 1989). Recruiting larger muscle masses (RHG to SLE and BIKE) stimulates more mechanoreceptors and increases afferent feedback. Therefore, the intermediate rise in PHC is most consistent with concurrent signaling from central command and mechanoreceptor feedback. Overall, these findings demonstrate that PHC is augmented during exercise; the experimental design cannot distinguish the relative contributions of neurally mediated factors, including central command and muscle afferent feedback. Therefore, the present data support that the transition from rest to exercise is associated with an increase in PHC.

### 4.6 Pre‐stimulus consistency

Ten minutes were provided between modalities for re‐instrumentation and recovery. Baseline PHC values did not differ between RHG and SLE, suggesting comparable pre‐exercise conditions. Although resting PHC during BIKE was greater than RHG, BIKE did not differ from SLE, and modality order was randomized, minimizing the likelihood of a systematic carryover effect. Resting HR did not differ between RHG and SLE, and pre‐V̇I was not different among any of the three modalities at rest, further supporting the adequate recovery. We previously showed elevated resting PHC when seated on a cycle ergometer compared to a chair, suggesting that anticipatory responses associated with the exercise modality may contribute to these differences rather than incomplete recovery from the previous exercise modality (Wright et al., 2024). The pre‐stimulus end‐tidal CO_2_ (pre‐P_ET_CO_2_) has an influence on PHC and therefore must be considered (Thompson et al., 2024). In the present study, both SLE and BIKE showed an elevated pre‐P_ET_CO_2_ during exercise compared to rest, creating a relatively hypercapnic environment prior to the stimulus. Relative hypercapnia would amplify the ventilatory response, but the pre‐stimulus cannot fully explain the observed increase (Thompson et al., 2025). In contrast, during RHG, the pre‐stimulus P_ET_CO_2_ was lower relative to the resting condition. This would create a hypocapnic environment, which would be anticipated to blunt the PHC response (Thompson et al., 2024). Even with the attenuation, RHG still showed a significant increase in PHC compared to rest, demonstrating that the exercise elated sensitization was a sufficient stimulus to elevate PHC despite lower pre‐P_ET_CO_2_. Both findings emphasize that while pre‐P_ET_CO_2_ must be considered as a modulating factor, it does not fully account for the augmentation or attenuation in chemosensitivity with exercise.

### 4.7 Methodological considerations

A limitation concerns the CO_2_ stimulus delivery during exercise with low‐ventilation conditions, (RHG and SLE). When tidal volumes are small, the two‐breath bolus may disperse across multiple breaths, partially mixing with room air in the tubing circuit and diminishing the effective ΔP_ET_CO_2_. In our cohort, a participant was excluded prior to data analysis for producing insufficient tidal volumes to fully clear the tubing during RHG/SLE, producing attenuated or absent PHC until BIKE, where larger tidal volumes were produced. The issue is easily identified by ensuring an adequate rise in P_ET_CO_2_. Secondly, it is not possible to directly quantify central command in humans. Because voluntary exercise inherently involves both central command and muscle mechanoreceptor feedback, the present design cannot fully distinguish their individual contributions. Third, afferent input from the peripheral chemoreceptors is also difficult to measure directly in exercising humans. Because both central command and peripheral chemoreceptor activity may change with the onset of exercise, we cannot determine the independent contribution of each mechanism to the observed PHC augmentation. Lastly, peripheral chemosensors respond to arterial carbon dioxide fluctuations and we used P_ET_CO_2_ as a surrogate. At rest in healthy individuals, P_ET_CO_2_ closely mirrors arterial CO_2_, but a gradient can develop especially with intense exercise (Swenson & Maren, 1978). To minimize this issue, we only utilized low‐intensity exercise which diminishes any potential gradient between end‐tidal and arterial CO_2_.

### 4.8 Future directions

In the present exploratory study, it is not possible to distinguish the relative roles of central command and muscle afferent feedback in contributing to the increase in PHC. Isolating these contributions will require an exercise modality that removes volitional effort from muscle activation itself. Future studies could use electrically evoked (non‐volitional) contractions to activate muscle without engaging central command. Comparing PHC during matched volitional and electrically evoked contractions would indicate whether central command is necessary for the augmentation in PHC or whether mechanoreceptor feedback alone is sufficient to reproduce it. Moreover, blocking muscle afferents while keeping motor drive via intrathecal fentanyl could be another experimental paradigm to disentangle the two mechanisms.

### 4.9 Conclusion

We found that each exercise modality, engaging different amounts of active muscle mass, elicited a rise in PHC from rest, but the augmentation did not entirely scale proportionally with muscle mass. Specifically, RHG produced the smallest increase in PHC, while SLE and BIKE evoked similar PHC responses despite the difference in the magnitude of recruited muscle mass. These findings suggest that the augmentation of PHC is more closely associated with the transition from rest to exercise than with further increases in workload or active muscle mass (Wright et al., 2024). Neurally mediated factors such as central command and muscle afferent feedback may contribute to this change; however, the present exploratory study cannot distinguish their relative roles.

## AUTHOR CONTRIBUTIONS

**Armando J. Aguero:** Conceptualization; data curation; formal analysis; investigation; methodology. **Connor J. Doherty:** Conceptualization; data curation; formal analysis; investigation; methodology. **Paolo B. Dominelli:** Conceptualization; data curation; formal analysis; funding acquisition; investigation; methodology; project administration; resources; supervision.

## FUNDING INFORMATION

The study was supported by a Discovery grant from NSERC (RGPIN‐2019‐04615) and an infrastructure grant from the Canada Foundation for Innovation (#38432).

## CONFLICTS OF INTEREST STATEMENT

No conflicts of interest, financial or otherwise, are declared by the authors.

## Data Availability

Data that support the findings will be made available upon reasonable request to the corresponding author.
